# Implementation of the COVID-19 laboratory testing certification program (CoLTeP), Zimbabwe, 2021

**DOI:** 10.11604/pamj.2022.42.204.34040

**Published:** 2022-07-14

**Authors:** Tendai Chipendo, Takudzwa Marembo, Clayton Munemo, Humphrey Chituri, Edwin Shumba, Donewell Bangure, Talkmore Maruta

**Affiliations:** 1 Africa Centres for Disease Control and Prevention, African Union Commission, Roosevelt Street (Old Airport), Addis Ababa, P.O. Box 3243, Addis Ababa, Ethiopia,; 2 Department of Medical Microbiology, Midlands State University Faculty of Medicine, Gweru, Zimbabwe,; 3 Ministry of Health and Child Care, Harare, Zimbabwe,; 4 African Society for Laboratory Medicine, Harare, Zimbabwe

**Keywords:** COVID-19, laboratory, certification program, Zimbabwe

## Abstract

The Africa Union (AU) Trusted Travel Initiative was introduced in 2021 to support Africa Union member states enhance their current health screening systems. Trusted Travel offers an online digital platform for the verification and authentication of COVID-19 results based on a collaborative effort across a network of participating COVID-19 testing laboratories. In this paper, we describe the certification process of laboratories to qualify for listing on the AU Trusted Travel platform as approved and recognized COVID-19 testing facilities. A checklist prepared from the ISO15189: 2012, ISO15190: 2020 and World Health Organization Laboratory Safety Manual, 4^th^ edition was used to audit laboratories. Approved auditors completed the audit checklist through reviewing laboratory documents and records, observing laboratory operations whilst asking open-ended questions to clarify documentation seen and observations made. A laboratory was recommended for certification after scoring at least 90%. Between May and September 2021, a total of 26 (19%) of the 134 medical laboratories authorized for SARS-CoV-2 testing had been audited for CoLTeP certification in Zimbabwe. The majority 16 (62%) attained 5 stars rating with 10 (38%) attaining 0-4 stars. Performance was highest in the area of test result and data management (mean score 93%, SD 9.1). The least performance of the laboratories was on the laboratory biosafety and biosecurity (mean score 73%, SD 17.0) and Quality Control and Assurance (mean score 71%, SD 15.0). There is need for laboratories to commit their resources to quality assurance programs and training of laboratory personnel in biosafety and biosecurity as part of continuous quality improvement.

## Introduction

Coronavirus disease 2019 (COVID-19) which was first reported on 31^st^ December 2019 from Wuhan, China was affirmed as a pandemic by the World Health Organisation (WHO) on 11^th^ March 2020 [1]. With 267,865,289 confirmed cases of COVID-19 and 5,285,88 deaths globally reported to WHO as of 12^th^ December 2021, the COVID-19 pandemic has had an enormous impact on the world [2]. In Africa over 8.8 million cases and 224,609 deaths have been reported as of 12 December 2021 [3]. After the first confirmed case on 20^th^ March 2020 in Zimbabwe, the number of cases and deaths rose to over 165,002 and 4,735, respectively as of 12^th^ December 2021 [4]. Diagnostic testing to identify individuals infected with Severe Acute Respiratory Syndrome coronavirus-2 (SARS-CoV-2) infection is crucial in the control of the COVID-19 pandemic. Firstly, efficient and timely testing is a vital prerequisite for early identification and reporting of COVID-19. This enables adequate contact tracing, isolation (of cases) and quarantine of contacts, which is critical in preventing transmission and slowing down the spread of SARS-CoV-2 [5]. Secondly, timely diagnosis facilitates early management of the disease to increase the recovery rate and lower mortality of COVID-19. Finally, testing provides accurate estimates of the presence and spread of SARS-CoV-2 in the population as well as monitoring of trends so as to inform the appearance of new waves, thus informing the need to put appropriate measures to slow spread [5]. To contain the pandemic, countries have also attempted to reduce the number of imported cases by implementing travel restrictions including requirement of international travellers to produce evidence of a negative SARS-CoV-2 test at points of entry [6]. However, as the risk grows, so does the critical need to strengthen the system and to harmonize cross-border COVID-19 requirements, thereby assuring the integrity of SARS-CoV-2 results presented.

The African Union (AU), Africa Centers for Disease Control and Prevention (CDC), African Society for Laboratory Medicine (ASLM) and other partners, working with PanaBIOS as technical leads introduced the African Union Trusted Travel Initiative to support AU member states harmonise and enhance their current health screening systems [7]. Trusted Travel offers an online digital platform for the verification and authentication of COVID-19 test results based on a collaborative effort across a network of participating COVID-19 testing laboratories and port health authorities. This platform allows for the detection of counterfeit travel documents and also enhances cross-border collaboration and confidence in COVID-19 results originating from other countries [8]. The platform further provides updated information on entry requirements and travel restrictions for participating states. In Zimbabwe, the trusted travel platform for the verification of COVID-19 certificates was launched on 23^th^ July 2021 [9]. Africa Union´s Trusted Travel Platform requires that there be a database of authorised laboratories certified to conduct COVID-19 testing that port health officials and other stakeholders can use to verify their authenticity [7]. African Society for Laboratory Medicine in collaboration with Africa CDC and other partners and Member States from Africa developed a COVID-19 Laboratory Testing Certification Program (CoLTeP) to ensure implementation of quality assurance, biosafety and biosecurity measures when testing for SARS-CoV-2. COVID-19 laboratory testing certification program is comprised of the following stakeholders: Ministries of Health focal person/office, applicant laboratories, COVID-19 laboratory testing certification auditors and ASLM [10]. Through standardized processes, CoLTeP evaluates a facility´s readiness and continued suitability to conduct quality COVID-19 testing safely. Where non conformities are identified, ASLM will assist in implementing corrective action and monitoring their effectiveness. This paper describes how the certification process of laboratories to qualify for listing on the AU Trusted Travel platform as an approved and recognized testing facility was implemented in Zimbabwe.

## Methods

To ensure sustainability and rapid rollout of the certification program, ASLM used locally trained and certified auditors to conduct the CoLTeP audits. The Ministry of Health and Child Care (MoHCC) focal person/office was responsible for identifying potential COVID-19 testing facilities, collection of required documents and submitting them to ASLM. Documents that were collected from the applicant laboratory included a completed application form, certificate of registration/authority to practice as a laboratory (if private laboratory) and a self-audit report conducted using the CoLTeP checklist. Upon review, a notification letter of enrolment with audit dates and locally assigned auditors was issued to the applicant laboratory through the MoHCC focal office/person.

**Auditor training, selection and deployment:** ASLM assisted the MoHCC with in-country training and certification of local auditors that were responsible for conducting CoLTeP audits. The training used a five-day standardized training curriculum that taught trainee auditors on the CoLTeP process, auditing skills, the CoLTeP checklist, the ISO 15189 standard and the WHO biosafety and biosecurity manual (2020) [9]. When a laboratory was approved through the MoHCC focal person to be audited for the CoLTeP, a team of in-country auditors was deployed with an overall lead auditor from ASLM who was responsible for coordination of all audits and submission of audits reports to ASLM.

**The CoLTeP checklist:** the CoLTeP checklist used for the auditing of COVID-19 testing laboratories was developed in May 2021 and is based on international standards including ISO 15189: 2012, ISO 15190: 2020 and the WHO Laboratory Biosafety Manual 4^th^ edition [10-12]. Each of the 57 questions on the checklist is allocated either two (2), three (3) or five (5) points depending on the complexity of the requirements of the question. The checklist is divided into five (5) sections which are: laboratory policies and procedures; laboratory testing capacity; Quality Control and Assurance; test result and data management and biosafety and biosecurity. A 0-5-star scale (Table 1) is used to rate laboratory performance at each audit. Only laboratories attaining 5-stars are recommended for certification as a COVID-19 testing facility and enrolled onto the AU Trusted Travel platform. The MoHCC and ASLM coordinates technical assistance for laboratories attaining 0-4 Stars and the laboratories are recommended to reapply for auditing after the raised non conformities have been addressed at least 3 months from date of last audit. Laboratories with 3-4 stars can submit evidence of closure of non-conformities electronically while the 0-2 stars require another visit by the auditor for a re-assessment.

**Table 1:** the CoLTeP checklist covering 5 sections, the points allocated to each section and the overall star rating system

Audit score sheet					
**Section**					**Total Points**
Section 1: laboratory policies and procedures					27
Section 2: laboratory testing capacity					29
Section 3: quality control and assurance					25
Section 4: test result and data management					17
Section 5: biosafety and biosecurity					32
**TOTAL**					130
**No Stars**	**1 Star**	**2 Stars**	**3 Stars**	**4 Stars**	**5 Stars**
(0-64 pts)	(65-77 pts)	(78-90pts)	(91-103 pts)	(104-116 pts)	(117-130 points)
< 50%	50-59%	60-69%	70-79%	80-89%	≥90%

Source: African Society for Laboratory Medicine

**Laboratory audit:** the standard audit process consisted of an opening meeting, the audit and a feedback meeting. The auditor´s key responsibilities included objective identification of areas of non-conformity and providing on-site technical assistance and recommendations for closing identified gaps. At the end of the audit, the audit teams discussed their findings, agreed on scores and developed a draft audit report with documented non-conformities and recommendations. During the debrief session, given either to the laboratory or hospital management, the auditors provided feedback on the overall performance of the audited laboratory and advocated for implementation of corrective actions identified during the audit. The lead auditor submitted the audit report to the laboratory which was agreed upon by the laboratory and the audit team by the end of the debrief meeting before submission to the ASLM secretariat. The final report was supported by a form agreeing to the report content and signed by the laboratory representative. The report was then submitted to the Independent Advisory Committee (IAC) at ASLM where it was reviewed to validate the audit findings [9].

## Results

**Figure 1:**
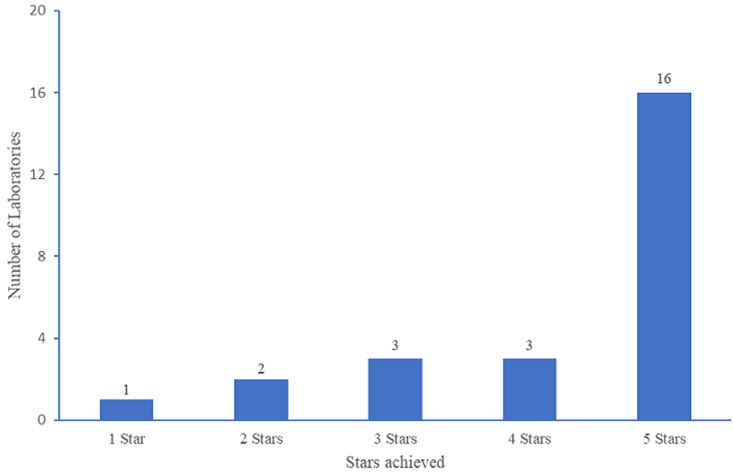
distribution of stars across the 26 CoLTeP-audited laboratories in Zimbabwe, May to September 2021

Between May and September 2021, 26 (19%) of the 134 private and MoHCC medical laboratories approved for SARS-CoV-2 testing in Zimbabwe had been audited for CoLTeP certification. Two (8%) of the 26 were Reference Laboratories, 1 (4%) was a research laboratory whilst the majority 22 (85%) were private-for-profit laboratories. Five (19%) of the audited laboratories were in the Bulawayo Province, 3 (12%) in the Midlands province, while the majority 18 (69%) were from Harare Province. There were no audited laboratories from Masvingo, Matebeleland south, Manicaland, Matebeleland north, Mashonaland east, Mashonaland west and Mashonaland central provinces. Of the 26 laboratories audited, 19 (73%) were performing real time Polymerase chain reaction (PCR) using open system whilst 7 (27%) were using cartridge based (closed) PCR systems. The distribution of stars across the 26 CoLTeP-audited laboratories between May and September 2021 is shown in Figure 1. The majority 16 (62%) attained 5 stars rating with 10 (38%) attaining 0-4 stars. The performance of the laboratories across the 5 sections of the CoLTeP checklist is shown in Table 2. The mean score for all the 26 laboratories was 82%, Standard Deviation (SD) 11.1. CoLTeP checklist Section 4 i.e. Test result and data management had the highest mean score of 93% (SD 9.1). The least performance of the laboratories was on biosafety and biosecurity (mean score 73%, SD 17.0) and Quality Control and Assurance (mean score 71, SD 15.0).

**Table 2:** summary of performance of the 26 laboratories across 5 sections of the CoLTeP checklist, May to September 2021

Section	Mean (%)	Standard deviation
Laboratory policies and procedures	83	13.3
Laboratory testing capacity	84	14.8
Quality control and assurance	71	15.0
Test result and data management	93	9.1
Biosafety and biosecurity	73	17.0
Final percentage score	82	11.1

**Figure 2:**
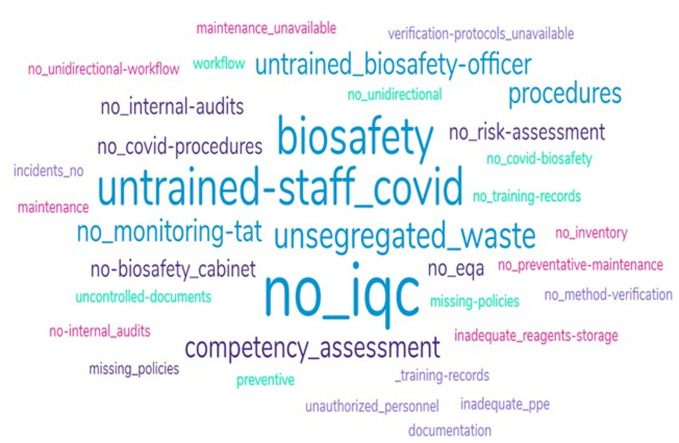
summary of word cloud analysis of non-conformities observed across the 26 CoLTeP audited laboratories, May to September 2021

**Summary of non-conformities observed:** a word cloud analysis of the non-conformities observed during the CoLTeP audits showed lack of internal quality controls (IQC) as the most frequently encountered non conformity. Other non-conformities encountered include lack of staff training on biosafety and biosecurity and laboratories not participating on COVID-19 External Quality Assessment (EQA) programs (Figure 2).

## Discussion

The CoLTeP tool can be used to improve the identified areas where corrective actions are required, as part of the laboratory´s continuous quality improvement. Other countries across the African continent including Malawi, Namibia and Zambia have adopted the CoLTeP tool to ensure compliance to standard requirements of testing for COVID-19 [13-15]. Eighty eight percent of the audited laboratories were private for profit whilst only 12% were MoHCC laboratories. In Zimbabwe, government run institutions are only testing those for diagnostic purposes being guided by the case definition and national testing strategy and testing is done for free. Those who are tested to be cleared for travelling across boarders are left to access such services in private-for-profit laboratories hence majority of the people who are tested at private for-profit laboratories are those who will be travelling across boarder. This could be the reason for more uptake of the CoLTeP by the private-for-profit sector as compared to government run institutions since the Africa Union Trusted Travel Platform´s main thrust is on providing verification of COVID-19 results for travellers. The three MoHCC run institutions at times perform tests for travellers who travel on government duties hence their much need to have applied for CoLTeP. Performance was highest in the area of test result and data management which looked at COVID-19 information management. The high performance in this area could be attributed to existence of electronic based resulting management system in all audited laboratories.

The least performance of the laboratories was observed on the CoLTeP section 3 (Quality Control and Assurance). The COVID-19 quality control and quality assurance section looked at practices including the use of internal quality controls, use of proficiency testing samples and the investigation and resolution of quality issues. This section forms part of the continual improvement activities inherent in a quality management system [16]. Lack of internal quality controls and external quality assessment was noted in a number of laboratories conducting COVID-19 testing. Since most of the audited laboratories were private-for-profit organizations, the issues of quality control and quality assurance brings with it an extra cost. This could have resulted in laboratories overlooking the issues of quality control as a way of cost cutting. Compromise in quality control issues has an impact on the credibility of the results produced by the laboratory and the CoLTeP program reaffirmed the importance of quality control. On the contrary, a study in Nigeria observed that quality control and quality assurance was more in private sector as compared to the public health sector [17]. Furthermore, lack of biosafety training for laboratory staff was identified as one of the major non conformities across the audited laboratories. Some laboratories performed general biosafety and biosecurity trainings that did not cover COVID-19 biosafety and biosecurity issues. At the beginning of the pandemic, the MoHCC with the support of Africa CDC and ASLM rolled out trainings on COVID-19 biosafety and biosecurity which was targeting capacitating public sector testing sites. Thus, laboratories within the MoHCC performed well on this aspect and most private laboratories had this as a non-conformity. Laboratory personnel are among the high-risk workers that have probable exposure to COVID-19 during sample collection and sample processing, hence the need for proper training on biosafety and biosecurity [18].

## Conclusion

Laboratories that enrolled and were audited in the CoLTeP complied with international requirements for quality, biosafety and biosecurity. Participation in quality assurance programs and training of laboratory personnel in biosafety and biosecurity in the context of COVID-19 remains a challenge.

**Recommendations:** only 26 laboratories were audited in the period under review and majority are still to participate in CoLTeP program. We therefore recommend mandatory participation in CoLTeP for all COVID-19 testing facilities to ensure quality results for both public health and travel purposes. Laboratories should commit their resources for infrastructure, human resources and training in laboratory quality improvement and biosafety and biosecurity as part of continuous quality improvement. Laboratories that have been certified for COVID-19 through the CoLTeP are encouraged to work towards ISO 15189 accreditation of the COVID-19 testing section. Private sector laboratories should engage more with the public health laboratories for trainings such as bio-safety and bio-security, waste management and quality management systems in COVID-19 testing laboratories.
